# Smoking Perceptions and Practice among Nursing Students in Kabupaten Kupang, Indonesia

**DOI:** 10.31557/APJCP.2019.20.6.1709

**Published:** 2019

**Authors:** Meksy S Pingak, Caroline L Miller

**Affiliations:** 1 *Community Health Centre of Oepoi, Department of Health, Kota Kupang, Indonesia, *; 2 *The University of Adelaide School of Public Health, *; 3 *South Australian Health and Medical Research Institute, Adelaide SA, Australia. *

**Keywords:** Nursing students, smoking, Indonesia

## Abstract

**Objective:**

Several studies have offered evidence of the importance of nursing-led interventions in smoking cessation. However, other studies have found that negative perceptions and smoking among nurses were barriers to them providing such interventions. The purpose of this study is to investigate smoking prevalence among nursing students and the demographic predictors of smoking, as well as perceptions about their roles with regard to smoking behaviour.

**Methods:**

A cross-sectional, self-administered, anonymous survey was conducted with all nursing students of the Maranatha School of Health Science in Indonesia. Smoking status, individual and familial characteristics including socio-economic status, and smoking cessation-related knowledge and attitudes were examined.

**Result:**

From the population of 313 students, 197 (62.9%) completed questionnaires were included in the analysis. The prevalence of current smoking for participants overall (25.9%) and males (52.4%) were similar to general population smoking rates in East Nusa Tenggara Province (25.9% overall and 52% for males) but lower than national rates (39% and 75.2%). However, the smoking rate among female participants (7%) was higher than national (2.9%) and regional (0.8%) female smoking rates. The majority of participants were aware of smoking health-related risk (87.3%) and supportive of giving smoking cessation advice (96.4%). In terms of seeing themselves as role models by not smoking at all, approximately 97% non-smokers agreed whereas only 60.8% of smokers agreed. Gender and being supportive of being role models by not smoking at all were significant predictors of the smoking status.

**Conclusion:**

This study suggests that smoking prevalence among nursing students is high. Despite most of the students having good smoking-related knowledge and having supportive attitudes towards providing smoking cessation services, high smoking prevalence is known to be an impediment to being effective in delivering cessation services. Personal smoking behaviour among nurses needs to be addressed to encourage critical nursing-led smoking cessation interventions.

## Introduction

Tobacco use causes approximately 6 million deaths annually, exceeding the lives lost due to HIV and AIDS, malaria and tuberculosis combined (WHO, 2015a). More than 1.1 billion smokers (80% of the world’s smokers) live in low- and middle-income countries (WHO, 2016). Tobacco use in these countries can create a “vicious circle” of poverty. The majority of smokers are from poor families and communities, and smoking related-illness and premature death deprives families of income, which in turn exacerbates poverty (WHO, 2008). 

Indonesia continues to contend with high prevalence of smoking, and unlike many nations, the prevalence of smoking in Indonesia has been increasing. Population smoking prevalence in Indonesia (among those aged > 15 years) rose from 32% in 2003 to 39% in 2015 (Indonesian Government, 2014; WHO, 2017a). Indonesia is ranked third in Asia and seventh in the world for overall population smoking prevalence. However, these averages mask very different smoking rates by gender. While female smoking prevalence is very low (only 2.9%) male smoking prevalence is high at 75.2%, with Indonesia ranking the highest in the world for male smoking (WHO 2015a; WHO, 2015b). The World Health Organization (2018) estimates that tobacco causes 225,720 deaths (14.7% of all deaths) in Indonesia each year. 

The prevalence of smoking is very likely to remain high in the future. Unlike many other countries, Indonesia does not have bans on direct tobacco advertising, there are limited smoke-free public places and workplaces, and there is a lack of smoking cessation support in health care facilities and communities (WHO, 2017a). Critically, cigarette prices remain very low in Indonesia, with cigarettes becoming more affordable between 2008 and 2016 (WHO, 2015a). The World Bank Group (2018) have forecast a future “public health and fiscal tsunami” from the rising rates of cancer and other non-communicable diseases and the resultant health and social costs if the high smoking prevalence in Indonesia remains.

The World Health Organization Framework Convention on Tobacco Control (WHO FCTC) is an evidence-based treaty negotiated under the auspices of the World Health Organization in 2003, developed in response to the global tobacco epidemic. The WHO FCTC and its guidelines provide the foundation for countries to implement and manage tobacco control via a comprehensive range of demand and supply side measures. Although 181 countries are parties to the WHO Convention on Tobacco Control, Indonesia did not ratify it until as recently as 2017 (United Nations, 2017), reflecting a history of low political commitment to implementation of tobacco control measures. 

The ‘MPOWER Package’ provides a country-level implementation guide to tobacco demand reduction strategies from the WHO FCTC (WHO, 2015a). An element of the MPOWER Package is to offer help for smokers to quit tobacco (WHO, 2008). Health professionals, including nurses, have been identified as fundamental to delivering cessation services, especially in low- and middle-income countries where affordability is a barrier to cessation services (Turk and El-Khoury, 2014). Nurse-delivered cessation interventions have been shown to be potentially effective to help enhance smokers’ motivation to quit or to quit completely, in a range of settings (Rice et al., 2017; Ergul and Temel, 2009; Venkatesh and Sinha, 2012).

Nurses can play a significant role in delivering smoking cessation interventions (Ozturk et al., 2011). Nurses comprise a large proportion of the clinical health care workforce and often have more time and consistent contact with patients compared with other health professionals. Nursing-led tobacco cessation interventions are also necessary, given the difficulties of routinely involving physicians in the services (Nichter et al., 2018). Moreover, nurses and nursing students have been shown to be viewed as role models by smokers and society (Ozturk et al., 2011). 

Despite the potential for the role, evidence indicates that smoking rates among nursing students can be high. Previous research in low- and middle-income countries found that smoking prevalence ranged from 3% in Iran to 31.3% in Chile (Smith, 2007; Henriques and Carvalho, 2008; Komu et al., 2009; Ozturk et al., 2011). Furthermore, studies among nurses have also indicated major deficits in the knowledge, skills and attitudes required to assess and counsel smoking patients effectively (Ozturk et al., 2011; Poreddi et al., 2015).

Although several studies have been conducted in low- and middle-income countries, no studies have been published on smoking among nursing students in Indonesia. In addition, a recent global survey of tobacco use involving nursing students across 40 countries did not include Indonesian nursing students (Warren et al., 2009). The purpose of this study is to investigate smoking prevalence among nursing students and its associated factors, including individual characteristics, socio-economic status, and smoking cessation-related knowledge and attitudes.

## Materials and methods

*Data collection and participant recruitment*

Data were collected via an anonymous, self-administered questionnaire. Prior to data collection, a pilot test of the questionnaire was carried out involving 15 nursing students from CHMK School of Health Science, in Kota Kupang, East Nusa Tenggara (ENT) Province, Indonesia. Participants were recruited through information distributed by the Head of School and class representatives. The survey was conducted on 14-15 March, 2018.

*Study population and variables*

The study population sampling frame was all registered undergraduate nursing students from the only health science school in Kabupaten Kupang, ENT Province, for the 2017/2018 academic year (N=313). ENT Province had the highest smoking prevalence (55.7%) among 33 provinces in Indonesia. Of 21 districts in the province, Kabupaten Kupang (74.6%) was in the top 5 for highest prevalence of tobacco smoking and chewing (Indonesian Government, 2015a; Indonesian Government 2015b). 

The questionnaire adopted several core questions from Global Health Professional Survey (WHO, 2017b). The dependent variable measured was smoking status with two categories: ‘current smoker’ and ‘non-smoker’ (including never smokers and ex-smokers). Current smoker was defined as those who smoked daily or occasionally (at least weekly, but not daily or less often than weekly) at the time of survey. Participants who had ever been a daily smoker in the past but had quit at the time of the survey were defined as ‘ex-smokers’. The independent variables measured were: gender, year of study, parental income, parental education, knowledge of health-related risk of smoking, knowledge about quit line (QL) and nicotine replacement therapy (NRT), and attitudes toward provision of smoking cessation interventions and role modelling smoking behaviour.

The measure of parental income was based on participants’ reports about their parents’ monthly income. The information was categorized into: 1) ‘very low’, if the income below provincial minimum wage (<IDR1,525,000), 2) ‘low’ (1,525,000-3,500,000), 3) ‘modest ‘(>3,500,000-6,000,000), or 4) ‘high’ (>6,000,000). Parental education was classified as: 1) no school, 2) very low (elementary school), 3) low (junior high school), 4) middle (senior high school), or 5) high (≥diploma degree). Knowledge of smoking related health risks was assessed by asking participants about their perceptions of 13 different smoking risks, including cancers, heart and lung function, fertility, and fetal development. Knowledge was categorized into five levels: 1) poor (being aware of ≤6 risk statements), 2) fair (7-8), 3) good (9-10), 4) very good (11-12), and 5) excellent (13). Respondents were asked to respond to attitudinal statements about provision of smoking cessation interventions and role modelling smoking behaviour, with a 5-point scale from “strongly agree” to “strongly disagree”. Participants’ responses were classified into three categories: 1) agree (strongly agree or agree), 2) neutral, and 3) disagree (strongly disagree or agree).

*Data analysis*

STATA version 14 was used for statistical analysis. Frequency distributions were used to describe participants’ smoking status and responses to the independent variables and prtest was applied to consider differences in proportions. Given that the study outcome was a binary variable and all predictors were ordered and unordered categorical variables, associations between the outcome and predictor variables were assessed using logistic regression (Tai and Machin, 2013). A two-tailed test of logistic regression with threshold for statistical significance of 0.05 was utilised for the analysis.

Stepwise elimination was the method of the multivariate analysis. All covariates with a p-value <0.25 from the bivariate analysis were selected for the first model. Covariates that had a p-value <0.05 in the first model were included into the second model. The process continued until only significant covariates were retained in the model. Finally, each variable not included within the first model was added one at a time with the two covariates retained earlier (Hosmer, 2013).

*Ethics*

The study was approved by the University of Adelaide Human Research Ethics Committee (no. H-2018-037). 

## Results

A total of 203 questionnaires were collected from nursing students. However, six were excluded due to incomplete answers. A final sample of 197 questionnaires (115 from females and 82 from males) was included in the analysis, corresponding to a response rate of 62.9%. 

*Prevalence of smoking*

In total, 25.9% (95% CI: 20.2-32.5) of nursing students sampled reported that they currently smoked (Table 1). Among the current smokers, 16.2% identified themselves as daily smokers and 9.7% as occasional smokers. Further, 24.4% of current smokers smoked factory-made cigarettes and 12.8% smoked cigar, pipe or any other tobacco products. Furthermore, 16.7% were ex-smokers (95%CI: 12.1-22.7). 

*Associated factors*

The prevalence of smoking by gender and year of study are presented in Table 1. The prevalence of current smoking for males (52.4%) was statistically higher than for female (7%) students (OR 14.7, 95%CI: 6.4-34.1, p= 0.000). Year of study did not predict current smoking status (OR 1.2, 95%CI: 0.9-1.7, p= 0.196). 

In general, the students came from low socio-economic status backgrounds. More than three in five of the students (65.3%) reported a very low level of parental income, below provincial minimum wage. Low parental education levels were prevalent (see Table 2), with 31.5% of students’ fathers and 36.0% of students’ mothers having only completed elementary school. No significant relationships were observed between smoking status and parental income (OR 0.8, 95%CI: 0.5-1.2, p= 0.330), father’s education (OR 0.9, 95%CI: 0.7-1.2, p= 0.504), or mother’s education (OR 0.8, 95%CI: 0.6-1.1, p= 0.147).

Overall, nursing students’ knowledge of smoking-related risks was moderate (Table 2). The majority of the students (87.3%) had either good (23.8%), very good (25.9%), or excellent (37.6%) awareness of the different harmful effects of tobacco smoking listed. Statistical analyses revealed no association with smoking status, indicating a similar level of the knowledge between non-smokers and smokers (OR 0.9, 95%CI: 0.7-1.2, p= 0.486).

Regarding cessation services, about 85% had heard of the QL and NRT. A similar proportion (85.3%) expressed agreement that they had the training and skills to give smoking cessation advice to patients. No significant associations were observed between smoking status and ever heard of the QL (OR 0.8, 95%CI: 0.4-1.9, p= 0.664), ever heard of NRT (OR 1.1, 95%CI: 0.9-1.3, p=0.530) and feeling confident in having training and skills (OR 1.5, 95%CI: 0.8-2.8, p=0.208). 

Data in Table 2 suggests that the majority of participants had positive attitudes towards provision of smoking cessation interventions. The majority of participants endorsed statements about nurses having a role in giving smoking cessation advice to patients (96.4%). Most participants reported feeling confident about asking about patients’ smoking behaviour (91.9%), and over half reported feeling confident in referring smoking patients to the QL (66.5%) or in encouraging the patients to use the NRT (66.5%). Confidence to ask about patients’ smoking behaviour was significantly related to smoking status (OR 2.6, 95% CI: 1.1-6.2, p= 0.026), with non-smokers expressing more confidence than smokers, but the other cessation advice variables were not related to smoking status. 

Overall 91.9% of participants agreed that nursing students should be a role model by not smoking in front of clients, and 87.3% thought that nursing students should not smoke at all. While non-smokers were more likely than smokers to support being role model, by not smoking in front of clients (OR 1.8, 95%CI: 1.0-3.3, p = 0.034) or by not smoking at all (OR 10.9, 95%CI :4.1-28.9, p= 0.000), smokers also expressed majority support (see Table 2).

Nine covariates with p-value <0.25 from the bivariate analysis were included into a model of multivariate logistic regression analysis. Only gender and being a role model by not smoking at all were significantly associated with current smoking behaviour. 

## Discussion

Smoking prevalence among nursing students overall (25.9%) and among male nursing students (52.4%) was similar to the general population in ENT Province (25.9% and 52% respectively) but lower than national rates (39% and 75.2%). However, smoking among female nursing students (7%) was higher than both national (2.9%) and regional (0.8%) female smoking rates (Indonesian Government, 2015b; WHO, 2017a). While largely reflective of local population smoking prevalence patterns, the high rates of smoking observed among nursing students is especially problematic given their potential future role in delivering nursing-led smoking cessation services; a significant component of comprehensive tobacco control efforts.

When compared to studies of nursing students from other low- and middle-income countries, observed smoking prevalence in this study was higher than that found in Iran (3%), Kenya (8.8%), and Turkey (19.6%) (Smith, 2007; Komu et al., 2009; Ozturk et al., 2011). In terms of other medical students, a survey undertaken in Malaysia, India, Pakistan, Nepal, and Bangladesh reported smoking prevalence of 13.1% (Sreeramareddy et al., 2010); lower than the rate observed in the present survey. These studies reinforce the finding that prevalence of smoking is high among Indonesian nursing students. 

In the present study, males were significantly more likely to smoke than females. The pattern of behaviour is consistent with smoking rates in the wider Indonesian population and is likely to be a reflection of Indonesian cultural norms. Social stigma is attached to women smoking in Indonesia, with smoking among women commonly considered to be inappropriate behaviour. This cultural norm appears to be present in many eastern countries. In the recent global survey of tobacco use among nursing students, Waren et al., (2009) found that smoking rates for females across 11 Asian countries were below 10%. By contrast, smoking prevalence was above 30% in 7 out of 10 European countries surveyed. 

Smoking prevalence across years of study was highest among participants in their fourth-year. This confirmed findings of a study in Spain (Gracia et al., 2007) yet is contrary to a survey in Turkey where smoking was more prevalent in first-year students than more senior students (Ozturk et al., 2011). The present study findings could be an indication of poor efficacy of smoking prevention programs in the school. In addition, there may be a perception that smoking is a stress reliever (Uchtenhagen, 2004) in the face of academic pressure and/or it could reflect the social influence of friends or families who smoked (Mony et al., 2010). 

Smoking status did not vary by parental income or education. This observation aligns with findings from a survey in Finland (Poutiainen et al., 2015). Previous research has reported that most smokers in low- and middle-income countries have insufficient income (Uchtenhagen, 2004; WHO, 2008). In the current study almost all participants (87.2%) were of either very low (65.3%) or low (21.9%) income. 

**Table 1:** Smoking Status According to Gender and Year of Study

			Current smokers				
	Current smoker	Non- smoker	Daily	Occasional	Cigarette smoker	Other tobacco use	Ex-smoker
	N (%)	N (%)	n (%)	n (%)	n (%)	n (%)	n (%)
All (N=197)	51 (25.9)	146 (74.1)	32 (16.2)	19 (9.7)	48 (24.4)	25 (12.8)	33 (16.7)
Female (n=115)	8 (7.0)	107 (93.0)	5 (4.4)	3 (2.6)	7 (6.1)	3 (2.7)	5 (4.4)
Male (n=82)	43 (52.4)	39 (47.6)	27 (32.9)	16 (19.5)	41 (50.0)	22 (26.8)	28 (34.2)
	OR 14.7, (95% CI: 6.4-34.1)						
	p* = 0.000						
Year of study							
1st year (n=26)	4 (15.4)	22 (84.6)	3 (11.5)	1 (3.9)	4 (15.4)	1 (3.9)	4 (15.4)
2nd year (n=55)	15 (27.3)	40 (72.7)	9 (16.4)	6 (10.9)	15 (27.3)	6 (11.1)	12 (21.8)
3rd year (n=63)	15 (23.8)	48 (76.2)	8 (12.7)	7 (11.1)	12 (19.1)	9 (14.5)	7 (11.1)
4th year (n=53)	17 (32.0)	36 (68.0)	12 (22.6)	5 (9.4)	17 (32.1)	9 (17.0)	10 (18.9)
	OR 1.2, (95% CI: 0.9-1.7)						
	p* = 0.196						

*Logistic regression, two sided p.value<0.05

**Table 2:** Current Smoking Status by Demographics and Attitudinal Variables

		N (%)	Current smoker				OR (95%CI)		P-value*	
			Yes (%)	No (%)						
Parental income		196 (100.0)	51 (26.0)	145 (74.0)			0.8 (0.5-1.2)		0.330	
	Very low	128 (65.3)	39 (76.5)	89 (61.4)						
	Low	43 (21.9)	6 (11.8)	37 (25.5)						
	Modest	19 (9.7)	3 (5.9)	16 (11.0)						
	High	6 (3.1)	3 (5.9)	3 (2.1)						
Father's education		197 (100.0)	51 (25.9)	146 (74.1)			0.9 (0.7-1.2)		0.504	
	No school	12 (6.1)	7 (13.7)	5 (3.4)						
	Very low	62 (31.5)	13 (25.5)	49 (33.6)						
	Low	36 (18.3)	7 (13.7)	29 (19.9)						
	Middle	49 (24.8)	16 (31.4)	33 (22.6)						
	High	38 (19.3)	8 (15.7)	30 (20.5)						
Mother's education		197 (100.0)	51 (25.9)	146 (74.1)			0.8 (0.6-1.1)		0.147	
	No school	11 (5.6)	6 (11.8)	5 (3.4)						
	Very low	71 (36.0)	19 (37.3)	52 (35.6)						
	Low	41 (20.8)	7 (13.7)	34 (23.3)						
	Middle	55 (27.9)	17 (33.3)	38 (26.0)						
	High	19 (9.7)	2 (3.9)	17 (11.6)						
Knowledge of smoking health- related risk		197 (100.0)	51 (25.9)	146 (74.1)			0.9 (0.7-1.2)		0.468	
	Poor	5 (2.5)	1 (2.0)	4 (2.7)						
	Fair	20 (10.2)	8 (15.7)	12 (8.2)						
	Good	47 (23.8)	11 (21.6)	36 (24.7)						
	Very good	51 (25.9)	13 (25.5)	38 (26.0)						
	Excelent	74 (37.6)	18 (35.3)	56 (38.4)						
Ever heard about the QL		197 (100.0)	51 (25.9)	146 (74.1)			0.8 (0.4-1.9)		0.664	
	No	31 (15.7)	9 (17.6)	22 (15.1)						
	Yes	166 (84.3)	42 (82.4)	124 (84.9)						
Ever heard about NRT		197 (100.0)	51 (25.9)	146 (74.1)			1.1 (0.9-1.3)		0.530	
	No	30 (15.2)	8 (15.7)	22 (15.1)						
	Yes	167 (84.8)	43 (84.3)	124 (84.9)						
Feeling confident in having training and skills to give smoking cessation advice to patients		197 (100.0)	51 (25.9)	146 (74.1)			1.5 (0.8-2.8)		0.208	
	Agree	168 (85.3)	42 (82.4)	126 (86.3)						
	Neutral	22 (11.2)	5 (9.8)	17 (11.6)						
	Disagree	7 (3.5)	4 (7.8)	3 (2.1)						
Nurses should give smoking cessation advice to patients		196 (100.0)	51 (26.0)	145 (74.0)			2.5 (0.8-8.0)		0.119	
	Agree	189 (96.4)	47 (92.2)	142 (97.9)						
	Neutral	5 (2.6)	3 (5.9)	2 (1.4)						
	Disagree	2 (1.0)	1 (2.0)	1 (0.7)						
Feeling confident to ask patients about their smoking behaviour		197 (100.0)	51 (25.9)	146 (74.1)			2.6 (1.1-6.2)		0.026	
	Agree	181 (91.9)	43 (84.3)	138 (94.5)						
	Neutral	13 (6.6)	6 (11.8)	7 (4.8)						
	Disagree	3 (1.5)	2 (3.9)	1 (0.7)						
Feeling confident to refer smoking patients to the QL		197 (100.0)	51 (25.9)	146 (74.1)			1.4 (0.8-2.2)		0.168	
	Agree	131 (66.5)	30 (58.8)	101 (69.2)						
	Neutral	49 (24.9)	15 (29.4)	34 (23.3)						
	Disagree	17 (8.6)	6 (11.8)	11 (7.5)						
		N (%)	Current smoker			OR (95%CI)		P-value*	
			Yes (%)		No (%)				
Feeling confident to encourage smoking patients to use the NRT		197 (100.0)	51 (25.9)		146 (74.1)	1.3 (0.8-1.9)		0.310	
	Agree	131 (66.5)	31 (60.8)		100 (68.5)				
	Neutral	41 (20.8)	12 (23.5)		29 (19.9)				
	Disagree	25 (12.7)	8 (15.7)		17 (11.6)				
Being role model by not smoking in front of clients		197 (100.0)	51 (25.9)		146 (74.1)	1.8 (1.0-3.3)		0.034	
	Agree	181 (91.9)	42 (82.4)		139 (95.2)				
	Neutral	4 (2.0)	4 (7.8)		0 (0.0)				
	Disagree	12 (6.1)	5 (9.8)		7 (4.8)				
Being role model by not smoking at all		197 (100.0)	51 (25.9)		146 (74.1)	10.9 (4.1-28.9)		0.000	
	Agree	172 (87.3)	31 (60.8)		141 (96.6)				
	Neutral	14 (7.1)	9 (17.6)		5 (3.4)				
	Disagree	11 (5.6)	11 (21.6)		0 (0.0)				

*Logistic regression, two sided p.value<0.05

Most nursing students had a high level of smoking-related knowledge. In the current study, 87% of respondents were aware of smoking-related health risks which is higher than has been found in a Kyrgyzstan study (74%) of health professional students (Brimkulov et al., 2017). Likewise, 84.8% of respondents had ever heard of NRT, which is higher than reported in Laos (37.1%; Sychareunt et al., 2013). About 85% of participants felt confident in their training and skills to deliver cessation advice, which is higher than nursing students in India (19.1%; Mony et al., 2010). High smoking-related health knowledge aligned with high participant agreement about having tobacco cessation training and skills, reflecting positively on the impact of smoking-related education in the nursing school. Smoking-related health knowledge is important for nurses delivering smoking cessation advice, particularly given there is often low community knowledge regarding the health risks associated with smoking in low- and middle-income countries (ITC Project et al., 2012), 

However, high smoking-related health knowledge did not appear to influence smoking among nursing students. While Lenz (2008) found that knowledge about the adverse health consequences of smoking was lower among nursing students who smoked than non-smokers, this was not observed in the present study. Knowledge of smoking related health risks, however, has been shown to be associated with confidence, which is needed to engage in delivery of smoking cessation interventions (Shishani et al., 2013).

Participants’ positive disposition towards delivering smoking cessation advice to patients (96.4%) was comparable with findings from a survey conducted by Sychareun et al., (2013) in Laos (95.3%). Participants in the present study were more positively disposed towards asking patients about their smoking behaviour (91.9%) than was observed among Indian nursing students (68.5%; Poreddi, 2015). When considering nursing students’ attitudes towards being role models by not smoking at all, smokers (60.8%) were less supportive than non-smokers (96.6%). This is unsurprising given the conflict between being a role model and their personal behaviours, but smokers in this study held less positive views than those of smoking health professional students’ perception in Laos (88%) (Sychareun et al., 2013). Negative perception about being role models could lead to negative impacts on smokers’ intention to take part in smoking cessation programs as well as motivation to quit smoking (Nichter et al., 2018).

Participants’ knowledge of and attitudes towards smoking cessation services were generally satisfactory. This creates potential to increase the likelihood of these students engaging smoking patients in cessation services and encouraging quitting when the students enter the workforce (Venkatesh and Sinha, 2012). However, the high smoking prevalence among nursing students may present as a major barrier to delivering cessation advice to future patients. Patients’ belief in tobacco related health advice will be reduced if they observe their cessation counselors smoking (Nichter et al., 2018). Therefore, it is important to reduce tobacco smoking among nursing students who may act as future cessation counsellors. Dedicated efforts to prevent nursing students from initiating smoking, to assist current smokers in quitting, and to instill a sense of responsibility as a role model to future patients should be incorporated into their curricular as early as first-year. 

The present study is not without limitations. The generalisability of the findings cannot be made to all health professional students as it only incorporates nursing students. In addition, this study did not collect additional information such as number of cigarettes smoked or family and friends’ tobacco consumption. Further research may be needed to consider factors influencing changes in smoking behaviour as students progress through their nursing degree. 

This study contributes to the knowledge of tobacco use among nursing students in Indonesia. It indicates that smoking prevalence among nursing students was high, consistent with the very high smoking rates in Indonesia more broadly. Therefore, despite knowledge and positive attitudes towards smoking cessation services expressed by nursing students, there is a need for nursing schools to proactively implement effective smoking prevention programs. If nursing students continue to smoke, this may prevent effective delivery of tobacco cessation services when entering the workforce.

## Funding

This study was funded by Indonesia Endowment Fund for Education (LPDP).
